# Resistance profile to antimicrobial agents in the main circulating bacteria isolated from acute periodontal and endodontic infections in Latin America (MICROBE- DENT): A systematic review protocol

**DOI:** 10.1097/MD.0000000000013158

**Published:** 2018-11-30

**Authors:** Flávia Casale Abe, Cristiane de Cássia Bergamaschi, Mabel Fernandes Figueiró, Luciane Cruz Lopes

**Affiliations:** a Department of Pharmaceutical Sciences, University of Sorocaba, Sorocaba, State of São Paulo; b Oswaldo Cruz Foundation, Fiocruz, Brasília, DF, Brazil.

**Keywords:** antibiotics, antimicrobial resistance, dentistry, endodontics, Latin America, periodontics

## Abstract

**Background:**

Antimicrobial resistance is currently considered the main risk to global health. A variety of microbial species have been isolated from endodontic and periodontal infections. However, clinical endodontic and periodontics bacterial isolates have not been sufficiently characterized with regard to their capacity for antibiotic resistance. We aim to assess the existing evidence to estimate the prevalence of the main antimicrobial resistance and multidrug resistant organisms in endodontics and periodontics and to describe their geographic distribution in Latin America.

**Methods:**

All types of designs and will be restricted to Latin American studies will be included in this systematic review. MEDLINE, Embase, CINAHL, BVS (LILACS, BBO - bvsalud.org), IBECS (bases.bireme.br), Google Scholar, Cochrane Central Register of Controlled Trials, and Web of Science databases will be searched from 2013 to December 31, 2018 for all types of study designs that report microbial infection in endodontics and periodontics and their resistance and that define the microbiological methods used to identify microorganisms. The selection of articles for inclusion will be performed by 2 reviewers using predefined eligibility criteria. The Cochrane and ROBINS-I risk of bias assessment tools will be used to assess the methodological quality of randomized control trials. The Newcastle–Ottawa scale will be used to assess the quality of methodology in observational studies. The overall quality of evidence will be assessed using Grading of Recommendations Assessment, Development and Evaluation (GRADE) using the same principles and domains applied in the quality assessment of prognostic studies. The heterogeneity of the findings will be assessed using both the χ^2^ test and the *I*^2^ statistic. Sensitivity analysis will be performed by subgroup analyses and meta-regression to investigate the effect of study-level characteristics, such as age, gender, and methodological quality score, whenever possible. Publication bias across studies will be evaluated by visual inspection of the funnel plots and Begg's test for the results covered in 10 or more studies.

**Results:**

The evidence derived by this study will inform best practices for patients with endodontic and periodontal problems receiving antimicrobial agents.

**Conclusion:**

Successful completion will significantly impact clinical practice and contribute to improved prescribing competency.

**Protocol registration:**

PROSPERO—CRD42018077810.

## Abstract 2

Supplemental Digital Content is available in the text

## 1 Introduction

Antimicrobial resistance is a natural phenomenon for the sake of the survival and maintenance of the species and is present in all geographic regions.^[1]^ It is a worldwide public health problem with few therapeutic options and a negative impact on patients infected by multidrug resistant organisms (MDROs).^[2]^ MDROs, according to the most widely used criteria in the literature,^[3]^ are labeled as such because of their in vitro resistance to more than one antimicrobial agent.

The antimicrobial resistance of anaerobes isolated from primary endodontic infections has increased in the last decade in the Brazilian population.^[4]^ Considering that the bacteria from root canals could potentially develop antimicrobial resistance, their capacity to form a biofilm may facilitate the dissemination of antimicrobial resistance by horizontal gene transfer.^[5]^

Infections caused by drug-resistant bacteria are associated with increased morbidity and mortality and increased costs. Clinically, patients with acute apical abscess experience mild-to-severe pain, swelling and even trismus. Systemic manifestations could occur, including fever, lymphadenopathy, malaise, headache, and nausea.^[6]^ Acute dental abscesses have caused serious complications and even death.^[6,7]^

The increasing rate of resistance of microorganisms to penicillins or other antibiotics has generated concern among health authorities in Latin America.^[8–10]^ It is more threatening when considering the very limited number of new antimicrobial agents that are in development.^[3]^

In 2004, a study intended to detect bacterial species from abscess samples collected in Oregon and Rio de Janeiro suggested that the differences found in the bacteria detected or cultured in the studies could be associated with the geographic location.^[11]^ It is estimated that, due to its size and alarming magnitude, the epidemiology of resistance may show remarkable geographical variability and rapid temporal evolution.

A systematic review including 7 studies that evaluated 374 patients from different countries worldwide revealed that antimicrobial resistance rates varied according to the previous use of antibiotics.^[12]^ However, the authors did not evaluate the risk of bias and disregarded the findings’ chronology.

The data generated by this search could also help managers of public health systems to make better decisions, in addition to serving as an educational tool for prescribers to acquire a greater understanding and awareness of the importance of the rational use of antimicrobials, in line with the recommendations of the World Health Organization.^[10]^

Therefore, the objectives of this systematic review are to estimate the prevalence of the main microbially resistance and multidrug resistance organisms, to analyze the time course tendencies of resistance and multidrug resistance, and to describe the geographic distribution of resistance and multidrug resistance organisms.

## 2 Systematic review question

What is the resistance profile to antimicrobial resistance in main circulating bacteria isolated from acute periodontal and endodontics infections in Latin America?

## 3 Methods

### 3.1 Standards

The systematic review will be performed according to the recommendations specified in the Cochrane Handbook for Interventional Reviews and reported according to the Preferred Reporting Items for Systematic Reviews and Meta-Analysis Protocols (PRISMA-P) statement (Additional file-1).

### 3.2 Protocol and Registration

We registered our review protocol with the International Prospective Register of Systematic Reviews https://www.crd.york.ac.uk/prospero/ (PROSPERO-CRD42018077810). Ethical approval is not required because this is a literature-based study.

### 3.3 Eligibility criteria

#### 3.3.1 Inclusion criteria

This systematic review will include all types of designs and will be restricted to Latin American studies meeting the following criteria: investigate microbial infection in endodontics and periodontics, report resistance to microbial infection in endodontics and periodontics, and discuss the microbiological methods used for the identification of microorganisms. Antimicrobial resistance is understood as the ability of a microorganism to resist the effects of an antimicrobial agent that previously could successfully treat the disease. Antimicrobial resistance will be defined as the resistance of an isolated pathogen to the antibiotic in question using a standardized antimicrobial susceptibility test model such as the agar diffusion test (Kirby–Bauer method) or other standard methods for determining the zone of inhibition or minimal inhibitory concentration (MIC) of the isolate. Additionally, we will include studies that detected the bacterial resistance genes of antibiotics by molecular techniques and those with samples collected from the buccal cavity (saliva, supragingival biofilm, or root canals with primary endodontic infections). MDROs, according to the most widely used criteria in the literature,^[3]^ are labeled as such because of their in vitro resistance to more than one antimicrobial agent.

##### 3.3.1.1 Participants

The studies should include patients with permanent dentition and endodontic and/ or periodontal microbial infection.

##### 3.3.1.2 Timing

The last 5 years (from 2013 to 31 December 2018)

##### 3.3.1.3 Language

There will be no restrictions based on language.

#### 3.3.2 Exclusion criteria

We will exclude *crossover* studies and those with incomplete data or information, studies in which data on microbial agents could not be isolated, primary studies or systematic reviews with the qualitative synthesis of information, therapeutic guides, guidelines, abstracts, conferences, books, book chapters, and methodological studies.

### 3.4 Measure outcomes

The main outcomes will be to find the prevalence of antibiotic resistance (with 95% confidence intervals), proportion of drug resistance transmitted and acquired, rate of failure during antibiotic treatment, percentage and the overall percentage of resistance for each antimicrobial agent.

### 3.5 Search methods for primary studies

We will not impose any language restrictions or publication status.

#### 3.5.1 Electronic searches

We will search in the following electronic databases, with no publication status restrictions: MEDLINE, Embase, CINAHL, Google Scholar, Cochrane Central Register of Controlled Trials, and Web of Science. The BVS (bvsalud.org) will be used to search for studies in different databases, such LILACS (lilacs.bvsalud.org), BBO (bases.bireme.br), and IBECS (bases.bireme.br). In *Portal de Periodicos* (periodicos.capes.gov.br), we will search for dentistry and oral sciences sources. For primary studies, we will search in ReBEC (http://www.ensaiosclinicos.gov.br), Clinicaltrials.gov and the WHO Register (who.int).

#### 3.5.2 Searching other resources

Additionally, we will use the website “bancodeteses.capes.gov.br” to identify dissertations in the field, and websites such as the Grey Literature Report (http://www.greylit.org) will be searched as grey literature. If necessary, the lead authors of the studies will be contacted for further information.

### 3.6 Search strategy

For the profile of the circulating agents of antimicrobial resistance in Latin America, the search strategy will be conducted individually with MeSH terms such as: resistance to antimicrobial drugs and endodontic and/or periodontal infections. The search strategy to be used is described in Additional file-2. This same strategy will be tailored for each database or library listed. This search strategy will be performed in cooperation with a research librarian.

### 3.7 Eligibility determination

Following a calibration exercise, peer reviewers will evaluate titles and abstracts, independently and in duplicate, according to the eligibility criteria. Covidence systematic review software (Veritas Health Innovation, Melbourne, Australia. Available at www.covidence.org) will be used to manage the screening among reviewers.

The full-text publications of articles selected as potentially eligible will be acquired. After a second calibration exercise, the same pairs of reviewers will independently apply the eligibility criteria to the potentially eligible full texts using standard forms.

Differences will be resolved by consensus among all reviewers. To exclude studies that published their results in more than one article (data replication), a reviewer will review all eligible articles and identify those with one or more in common authors. In case of publication of data from the same cohort, the article with the most complete data will be used.

To evaluate the concordance of the selection for the full text, the Kappa test will be used. Kappa values between 0.40 and 0.59 will be considered to represent weak agreement, between 0.60 and 0.74 to represent intermediate agreement, and 0.75 or more to represent excellent agreement. Reviewers will use a standardized, pretested data extraction form with instructions on how to extract them. For articles published only in summary or articles that have important information missing, complete information on the methods and results will be obtained by contacting the authors.

### 3.8 Data extraction

Two reviewers, in pairs and independently, will be calibrated by extracting at least 3 articles and then coming to a consensus. This procedure should occur until the reviewers are able to extract the data.

Data on the patient's nosological status (type of infection, diagnosis), including the number of subjects included in the study, the description of recruitment, city, location, date of the research, exposure to antibiotics, sample size, type (saliva, supra-gingival biofilm, root canal with primary endodontic infection, etc.), methods of determining sample size, conflicts of interest, biological material used, methods used to measure results (type of medium of culture, type of collection, etc.), antimicrobial agents tested, number of bacterial lines and number of resistant species, will be collected from all studies.

The total percentage of an antimicrobial agent will be calculated for each study, regardless of the bacterial species tested.

The overall percentage resistance for each antimicrobial agent tested will be the average between the total number of resistant isolates and the total number of isolates evaluated. Microbial isolates with an intermediate profile will be considered susceptible to the antimicrobial agent.^[13]^

### 3.9 Risk of bias in individual studies

The risk assessment of bias will be independently assessed by at least 2 reviewers in duplicate using the instrument for nonrandomized studies by Cochrane (Collaboration tool for assessing the risk of bias—ROBINS-I)^[14]^ and considering specific tools for prevalence studies.^[15]^ For observational studies, the Newcastle–Ottawa quality scale for cohort studies will be used. If differences are observed, they will be resolved by consensus among all reviewers. Incomplete results will be stipulated as having a low risk of bias, with a loss of follow-up of <10%.

Two reviewers will independently evaluate the quality of each study included, and any disagreement will be resolved by consensus or by one arbitrator to judge unresolved disagreements.

### 3.10 Confidence in pooled estimates of effect

The overall quality of evidence will be assessed using Grading of Recommendations Assessment, Development and Evaluation (GRADE) using the same principles and domains applied in the quality assessment of prognostic studies.^[16]^

We will perform subgroup analysis for geographic area (country) and by dental specialty (endodontics and periodontics). If possible, we will also perform subgroup analyses for age, gender, and antimicrobial class. Analysis will be performed using R, V.3.2.3, and the meta V.4.3-2 and metaphor packages.

### 3.11 Data synthesis

The results will be analyzed separately according to the study design, and when possible, will be described qualitatively in tables of evidence. A descriptive summary will be created to determine the amount of evidence found and the variation between studies. The data will be grouped by microbial agents and antibiotic agents.

Statistical analysis of data (meta-analysis) will only be performed if appropriate data is found. The random-effects model will be used to calculate the pooled prevalence and corresponding 95% CI. If possible, the prevalence of the main circulating agents of antimicrobial resistance in periodontal and endodontic infections in Latin America will be adjusted, considering the population of each Latin American country.

The 95% predictive distribution, that is, the probabilistic interval of the realization of new studies to be carried out in Latin America, will be calculated.

The heterogeneity of the findings will be assessed using both the χ^2^ test and the *I*^2^ statistic. Sensitivity analysis will be performed by subgroup analyses and meta-regression to investigate the effect of study-level characteristics, such as age, gender and methodological quality score, whenever possible. Publication bias across studies will be evaluated by visual inspection of the funnel plots and Begg's test for the results covered in 10 or more studies.

### 3.12 Ethics and dissemination

No ethical approval is required as no primary, personal or confidential data are being collected in this study. Successful completion will significantly impact clinical practice and contribute to improved prescribing competency. This will inform best practices for patients with endodontic and periodontal problems receiving antimicrobial agents. The results of this study will be published in a peer-reviewed journal and presented at conferences.

## 4 Discussion

Our review will assess the existing evidence to estimate the prevalence of the main antimicrobial resistance and MDROs in endodontics and periodontics and to describe their geographic distribution in Latin America.

The data generated by this search could also help managers of public health systems to make better decisions, in addition to serving as an educational tool for prescribers to acquire a greater understanding and awareness of the importance of the rational use of antimicrobials, in line with the recommendations of the World Health Organization.^[10]^

The findings will be disseminated to national and international scientific sessions and published in a peer-reviewed journal. Successful completion will significantly impact on clinical practice and contribute to improve prescribing competency. This will inform best practice of patients with endodontic and periodontal problem receiving antimicrobial agents, and help facilitate evidence-based shared care decision-making. This study will also identify key areas for future research.

### 4.1 Strengths and limitations of this study

- To our knowledge, this is the first systematic review protocol that has attempted to evaluate the prevalence of antimicrobial resistance in the areas of endodontics and periodontics in Latin America.
- This protocol was written according to the PRISMA-P guidelines, and the review will be written using a standardized methodology with a full bibliographic search, study selection, data extraction, and bias risk assessment performed by 2 independent researchers.
- The chosen time period appears short (5 years) but represents the time necessary to verify changes in the profile of resistance.
- Using all types of designs and limiting the studies to Latin American research in the area of dentistry, where there is a clear lack of high-quality trials, will increase the internal validity.

## Author contributions

FCA is the principal investigator and wrote the protocol. MFF wrote the search strategy. The guarantor of the review is LCL. FCA and LCL will individually perform the abstract extraction and critique the literature, and CCB will be the third reviewer. CCB provided insight on the epidemiological aspects of the review and helped draft the manuscript. FCA, CCB, MFF and LCL advised on background and revised the manuscript. All authors approve the final version and take responsibility for its content.

**Conceptualization:** Flavia Casale Abe, Luciane Cruz Lopes.

**Methodology:** Flavia Casale Abe, Cristiane de Cassia Bergamaschi, Mabel Fernandes Figueiro, Luciane Cruz Lopes.

**Project administration:** Luciane Cruz Lopes.

**Supervision:** Luciane Cruz Lopes.

**Writing – original draft:** Flavia Casale Abe, Cristiane de Cassia Bergamaschi.

**Writing – review & editing:** Luciane Cruz Lopes.

## Supplementary Material

Supplemental Digital Content

## References

[1] D’Costa VM King CE Kalan L Antibiotic resistance is ancient. Nature 2011;477:457–61. 21881561 10.1038/nature10388

[2] Laxminarayan R Duse A Wattal C Antibiotic resistance—the need for global solutions. Lancet Infect Dis 2013;13:1057–98. 24252483 10.1016/S1473-3099(13)70318-9

[3] Magiorakos AP Srinvasan A Carey RB Multidrug-resistant, extensively drug-resistant and pandrug-resistant bacteria: an international expert proposal for interim standard definitions for acquired resistance. Clin Microbiol Infect 2012;18:268–81. 21793988 10.1111/j.1469-0691.2011.03570.x

[4] Gomes BPFA Jacinto RC Montagner F Analysis of the antimicrobial susceptibility of anaerobic bacteria isolated from endodontic infections in Brazil during a period of nine years. J Endod 2011;37:1058–62. 21763894 10.1016/j.joen.2011.05.015

[5] Al-Ahmad A Ameen H Pelz K Antibiotic resistance and capacity for biofilm formation of different bacteria isolated from endodontic infections associated with root-filled teeth. J Endod 2014;40:223–30. 24461408 10.1016/j.joen.2013.07.023

[6] Green AW Flower EA New NE Mortality associated with odontogenic infection!. Br Dent J 2001;190:529–30. 11411886 10.1038/sj.bdj.4801024

[7] Robertson D Smith AJ The microbiology of the acute dental abscess. J Med Microbiol 2009;58:155–62. 19141730 10.1099/jmm.0.003517-0

[8] Baumgartner JC Xia T Antibiotic susceptibility of bacteria associated with endodontic abscesses. J Endod 2003;29:44–7. 12540219 10.1097/00004770-200301000-00012

[9] File TM Solomkin JS Cosgrove SE Strategies for improving antimicrobial use and the role of antimicrobial stewardship programs. Clin Infect Dis 2011;53 suppl 1:S15–22. 21795724 10.1093/cid/cir364

[10] World Health Organization (WHO). Antimicrobial resistance: a manual for developing national action plans, 2016 Available at: http://apps.who.int/iris/bitstream/10665/204470/1/9789241549530_eng.pdf?ua=1 Accessed January 2018.

[11] Baumgartner JC Siqueira JF Jr Xia T Geographical differences in bacteria detected in endodontic infections using polymerase chain reaction. J Endod 2004;30:141–4. 15055430 10.1097/00004770-200403000-00004

[12] Lang PM Jacinto RC Dal Pizzol TS Resistance profiles to antimicrobial agents in bacteria isolated from acute endodontic infections: systematic review and meta-analysis. Int J Antimicrob Agents 2016;48:467–74. 27742205 10.1016/j.ijantimicag.2016.08.018

[13] Clinical and Laboratory Standards. Methods for Antimicrobial Susceptibility Testing of Anaerobic Bacteria; approved Standard M11-A8. Available at: http://shop.clsi.org/site/Sample_pdf/M11A8_sample.pdf: Clinical and Laboratory Standards Institute, 2012; 32: 1-39.

[14] Sterne JA Hernán MA Reeves BC ROBINS-I: a tool for assessing risk of bias in non-randomised studies of interventions. BMJ 2016;355:i4919. 27733354 10.1136/bmj.i4919 PMC5062054

[15] Loney PL Chambers LW Bennett KJ Critical appraisal of the health research literature: prevalence or incidence of a health problem. Chronic Dis Can 2000;19:1–1. http://www.collectionscanada.gc.ca/webarchives/20071212045422/http://www.phac-aspc.gc.ca/publicat/cdic-mcc/19-4/e_e.html 10029513

[16] Iorio A Spencer FA Falavigna M Use of GRADE for assessment of evidence about prognosis: rating confidence in estimates of event rates in broad categories of patients. BMJ 2015;350:h870. 25775931 10.1136/bmj.h870

