# Supplementary material for: Resistance profile to antimicrobial agents in the main circulating bacteria isolated from acute periodontal and endodontic infections in Latin America (MICROBE- DENT): A systematic review protocol
Source: Medicine (Baltimore). 2018 Nov 30;97(48):e13158. doi: 10.1097/MD.0000000000013158 (PMC6283129; doi:10.1097/MD.0000000000013158)
Supplement: Supplemental Digital Content [file medi-97-e13158-s001.pdf]

PRISMA-P (Preferred Reporting Items for Systematic review and Meta-Analysis Protocols) 2015 checklist: recommended items to address in a systematic review protocol*

Section and topic	Item No	Checklist item	Page on text
**ADMINISTRATIVE INFORMATION**			
Title: **Resistance profile to antimicrobial agents in main circulating bacteria isolated from acute Periodontal and Endodontics infections in Latin America (MICROBE-DENT): a systematic review protocol.**			
Identification	1a	Identify the report as a protocol of a systematic review	1
Update	1b	If the protocol is for an update of a previous systematic review, identify as such	Not applicable
Registration	2	If registered, provide the name of the registry (such as PROSPERO) and registration number	3
Authors:			
Contact	3a	Provide name, institutional affiliation, e-mail address of all protocol authors; provide physical mailing address of corresponding author	1
Contributions	3b	Describe contributions of protocol authors and identify the guarantor of the review	11
Amendments	4	If the protocol represents an amendment of a previously completed or published protocol, identify as such and list changes; otherwise, state plan for documenting important protocol amendments	Not applicable
Support:			
Sources	5a	Indicate sources of financial or other support for the review	11
Sponsor	5b	Provide name for the review funder and/or sponsor	Not applicable
Role of sponsor or funder	5c	Describe roles of funder(s), sponsor(s), and/or institution(s), if any, in developing the protocol	11
**INTRODUCTION**			
Rationale	6	Describe the rationale for the review in the context of what is already known	3-5
Objectives	7	Provide an explicit statement of the question(s) the review will address with reference to participants, interventions, comparators, and outcomes (PICO)	5
**METHODS**			
Eligibility criteria	8	Specify the study characteristics (such as PICO, study design, setting, time frame) and report characteristics (such as years considered, language, publication status) to be used as criteria for eligibility for the review	5-6

Information sources	9	Describe all intended information sources (such as electronic databases, contact with study authors, trial registers or other grey literature sources) with planned dates of coverage	6-7
Search strategy	10	Present draft of search strategy to be used for at least one electronic database, including planned limits, such that it could be repeated	7 and additional file 2
Study records:			
Data management	11a	Describe the mechanism(s) that will be used to manage records and data throughout the review	7-8
Selection process	11b	State the process that will be used for selecting studies (such as two independent reviewers) through each phase of the review (that is, screening, eligibility and inclusion in meta-analysis)	7-8
Data collection process	11c	Describe planned method of extracting data from reports (such as piloting forms, done independently, in duplicate), any processes for obtaining and confirming data from investigators	8
Data items	12	List and define all variables for which data will be sought (such as PICO items, funding sources), any pre-planned data assumptions and simplifications	8
Outcomes and prioritization	13	List and define all outcomes for which data will be sought, including prioritization of main and additional outcomes, with rationale	Not applicable
Risk of bias in individual studies	14	Describe anticipated methods for assessing risk of bias of individual studies, including whether this will be done at the outcome or study level, or both; state how this information will be used in data synthesis	8-9
Data synthesis	15a	Describe criteria under which study data will be quantitatively synthesised	9-10
	15b	If data are appropriate for quantitative synthesis, describe planned summary measures, methods of handling data and methods of combining data from studies, including any planned exploration of consistency (such as I^2, Kendall's τ)	9-10
	15c	Describe any proposed additional analyses (such as sensitivity or subgroup analyses, meta-regression)	9-10
	15d	If quantitative synthesis is not appropriate, describe the type of summary planned	Not applicable
Meta-bias(es)	16	Specify any planned assessment of meta-bias(es) (such as publication bias across studies, selective reporting within studies)	8-9
Confidence in cumulative evidence	17	Describe how the strength of the body of evidence will be assessed (such as GRADE)	9

*** It is strongly recommended that this checklist be read in conjunction with the PRISMA-P Explanation and Elaboration (cite when available) for important clarification on the items. Amendments to a review protocol should be tracked and dated.** **The copyright for PRISMA-P (including checklist) is held by the PRISMA-P Group and is distributed under a Creative Commons Attribution Licence 4.0.**

From: Shamseer L, Moher D, Clarke M, Ghersi D, Liberati A, Petticrew M, Shekelle P, Stewart L, PRISMA-P Group. Preferred reporting items for systematic review and meta-analysis protocols (PRISMA-P) 2015: elaboration and explanation. BMJ. 2015 Jan 2;349(jan02 1):g7647.

Database: OVID Medline Epub Ahead of Print, In-Process & Other Non-Indexed Citations, Ovid MEDLINE(R) Daily and Ovid MEDLINE(R) 1946 to Present
Search Strategy:
----
1 endodontics.mp. or exp ENDODONTICS/
2 periodontal disease.mp. or exp Periodontal Diseases/
3 Disease, Periodontal.mp. or exp Periodontal Diseases/
4 Diseases, Periodontal.mp. or exp Periodontal Diseases/
5 Parodontosis.mp. or Periodontal Diseases/
6 Parodontoses.mp. or exp Periodontal Diseases/
7 Pyorrhea Alveolaris.mp. or exp Periodontal Diseases/
8 Periodontitis.mp. or exp PERIODONTITIS/
9 aggressive periodontitis.mp. or exp Aggressive Periodontitis/
10 periapical periodontitis.mp. or exp Periapical Periodontitis/
11 Periodontitides.mp. or exp Periodontitis/
12 Pericementitis.mp. or exp Periodontitis/
13 Pericementitides.mp. or exp Periodontitis/
14 dental pulp disease.mp. or exp Dental Pulp Diseases/
15 Dental Pulp Diseases.mp. or exp Dental Pulp Diseases/
16 Pulp Diseases, Dental.mp. or exp Dental Pulp Diseases/
17 Diseases, Dental Pulp.mp. or exp Dental Pulp Diseases/
18 Pulp Disease, Dental.mp. or exp Dental Pulp Diseases/
19 Dental Pulp Disease.mp. or exp Dental Pulp Diseases/
20 Disease, Dental Pulp.mp. or exp Dental Pulp Diseases/
21 exp Periapical Abscess/ or Periapical Abscess.mp.
22 Dentoalveolar Abscess, Apical.mp. or exp Periapical Abscess/
23 Abscess, Apical Dentoalveolar.mp. or exp Periapical Abscess/
24 Abscesses, Apical Dentoalveolar.mp. or exp Periapical Abscess/
25 Apical Dentoalveolar Abscess.mp. or exp Periapical Abscess/
26 Apical Dentoalveolar Abscesses.mp. or exp Periapical Abscess/
27 Dentoalveolar Abscesses, Apical.mp. or exp Periapical Abscess/
28 Periodontitis, Apical, Suppurative.mp. or exp Periapical Abscess/
29 Periapical Periodontitis, Suppurative.mp. or exp Periapical Abscess/
30 Periapical Periodontitides, Suppurative.mp. or exp Periapical Abscess/
31 Periodontitides, Suppurative Periapical.mp. or exp Periapical Abscess/
32 Periodontitis, Suppurative Periapical.mp. or exp Periapical Abscess/
33 Suppurative Periapical Periodontitides.mp. or exp Periapical Abscess/
34 Suppurative Periapical Periodontitis.mp. or exp Periapical Abscess/
35 Alveolar Abscess, Apical.mp. or exp Periapical Abscess/
36 Abscess, Apical Alveolar.mp. or exp Periapical Abscess/
37 Abscesses, Apical Alveolar.mp. or exp Periapical Abscess/
38 Alveolar Abscesses, Apical.mp. or exp Periapical Abscess/
39 Apical Alveolar Abscess.mp. or exp Periapical Abscess/
40 Apical Alveolar Abscesses.mp. or exp Periapical Abscess/
41 Abscess, Periapical.mp. or exp Periapical Abscess/
42 Abscesses, Periapical.mp. or exp Periapical Abscess/
43 Periapical Abscesses.mp. or exp Periapical Abscess/
44 Periodontal Abscess.mp. or exp Periodontal Abscess/

45 Periodontal Abscesses.mp. or exp Periodontal Abscess/
46 Abscess, Periodontal.mp. or exp Periodontal Abscess/
47 Abscesses, Periodontal.mp. or exp Periodontal Abscess/
48 Periodontal Abscesses.mp. or exp Periodontal Abscess/
49 dental pulp cavity.mp. or exp Dental Pulp Cavity/
50 Cavity, Dental Pulp.mp. or exp Dental Pulp Cavity/
51 Pulp Cavities, Dental.mp. or exp Dental Pulp Cavity/
52 Pulp Cavity, Dental.mp. or exp Dental Pulp Cavity/
53 Cavities, Dental Pulp.mp. or exp Dental Pulp Cavity/
54 Dental Pulp Cavities.mp. or exp Dental Pulp Cavity/
55 dental care.mp. or exp Dental Care/
56 Care, Dental.mp. or exp Dental Care/
57 Pulp Chamber.mp. or exp Dental Pulp Cavity/
58 Chamber, Pulp.mp. or exp Dental Pulp Cavity/
59 Chambers, Pulp.mp. or exp Dental Pulp Cavity/
60 Pulp Chambers.mp. or exp Dental Pulp Cavity/
61 Pulp Canal.mp. or exp Dental Pulp Cavity/
62 Canal, Pulp.mp. or exp Dental Pulp Cavity/
63 Canals, Pulp.mp. or exp Dental Pulp Cavity/
64 Pulp Canals.mp. or exp Dental Pulp Cavity/
65 Canal, Root.mp. or exp Dental Pulp Cavity/
66 Canals, Root.mp. or exp Dental Pulp Cavity/
67 1 or 2 or 3 or 4 or 5 or 6 or 7 or 8 or 9 or 10 or 11 or 12 or 13 or 14 or 15 or 16 or 17 or 18 or 19 or 20 or 21 or 22 or 23 or 24 or 25 or 26 or 27 or 28 or 29 or 30 or 31 or 32 or 33 or 34 or 35 or 36 or 37 or 38 or 39 or 40 or 41 or 42 or 43 or 44 or 45 or 46 or 47 or 48 or 49 or 50 or 51 or 52 or 53 or 54 or 55 or 56 or 57 or 58 or 59 or 60 or 61 or 62 or 63 or 64 or 65 or 66
68 drug resistance, Microbial.mp. or exp Drug Resistance, Microbial/
69 Drug Resistances, Microbial.mp. or exp Drug Resistance, Microbial/
70 Drug Resistances, Microbial.mp. or exp Drug Resistance, Microbial/
71 Antimicrobial Drug Resistances.mp. or exp Drug Resistance, Microbial/
72 Antibiotic Resistance, Microbial.mp. or exp Drug Resistance, Microbial/
73 Antibiotic Resistance.mp. or exp Drug Resistance, Microbial/
74 Resistance, Antibiotic.mp. or exp Drug Resistance, Microbial/
75 antifungal drug resistance.mp. or exp Drug Resistance, Fungal/
76 Resistance, Antifungal Drug.mp. or exp Drug Resistance, Fungal/
77 Antibiotic Resistance, Fungal.mp. or exp Drug Resistance, Fungal/
78 microbial sensitivity tests.mp. or exp Microbial Sensitivity Tests/
79 Microbial Sensitivity Test.mp. or exp Microbial Sensitivity Tests/
80 Sensitivity Test, Microbial.mp. or exp Microbial Sensitivity Tests/
81 Sensitivity Tests, Microbial.mp. or exp Microbial Sensitivity Tests/
82 Test, Microbial Sensitivity.mp. or exp Microbial Sensitivity Tests/
83 Tests, Microbial Sensitivity.mp. or exp Microbial Sensitivity Tests/
84 Drug Sensitivity Assay, Microbial.mp. or exp Microbial Sensitivity Tests/
85 Antimicrobial Susceptibility Breakpoint Determination.mp. or exp Microbial Sensitivity Tests/
86 Breakpoint Determination, Antimicrobial Susceptibility.mp. or exp Microbial Sensitivity Tests/
87 Virus Drug Sensitivity Tests.mp. or exp Microbial Sensitivity Tests/
88 Viral Drug Sensitivity Tests.mp. or exp Microbial Sensitivity Tests/

89 Breakpoint Determination, Antibacterial Susceptibility.mp. or exp Microbial Sensitivity Tests/
90 Antibacterial Susceptibility Breakpoint Determination.mp. or exp Microbial Sensitivity Tests/
91 Fungus Drug Sensitivity Tests.mp. or exp Microbial Sensitivity Tests/
92 Fungal Drug Sensitivity Tests.mp. or exp Microbial Sensitivity Tests/
93 Minimum Inhibitory Concentration.mp. or exp Microbial Sensitivity Tests/
94 Concentration, Minimum Inhibitory.mp. or exp Microbial Sensitivity Tests/
95 Inhibitory Concentration, Minimum.mp. or exp Microbial Sensitivity Tests/
96 Inhibitory Concentrations, Minimum.mp. or exp Microbial Sensitivity Tests/
97 Minimum Inhibitory Concentrations.mp. or exp Microbial Sensitivity Tests/
98 Antibiogram.mp. or exp Microbial Sensitivity Tests/
99 Antibiograms.mp. or exp Microbial Sensitivity Tests/
100 Bacterial Sensitivity Tests.mp. or exp Microbial Sensitivity Tests/
101 Tests, Bacterial Sensitivity.mp. or exp Microbial Sensitivity Tests/
102 Sensitivity Tests, Bacterial.mp. or exp Microbial Sensitivity Tests/
103 Test, Bacterial Sensitivity.mp. or exp Microbial Sensitivity Tests/
104 Bacterial Sensitivity Test.mp. or exp Microbial Sensitivity Tests/
105 Sensitivity Test, Bacterial.mp. or exp Microbial Sensitivity Tests/
106 68 or 69 or 70 or 71 or 72 or 73 or 74 or 75 or 76 or 77 or 78 or 79 or 80 or 81 or 82 or 83 or 84 or 85 or 86 or 87 or 88 or 89 or 90 or 91 or 92 or 93 or 94 or 95 or 96 or 97 or 98 or 99 or 100 or 101 or 102 or 103 or 104 or 105
107 67 and 106

****
